# Current Clinical Strategies of Pancreatic Cancer Treatment and Open Molecular Questions

**DOI:** 10.3390/ijms20184543

**Published:** 2019-09-13

**Authors:** Maximilian Brunner, Zhiyuan Wu, Christian Krautz, Christian Pilarsky, Robert Grützmann, Georg F. Weber

**Affiliations:** Department of General and Visceral Surgery, Friedrich Alexander University, Krankenhausstraße 12, 91054 Erlangen, Germany; Maximilian.Brunner@uk-erlangen.de (M.B.); zhiyuan.wu@fau.de (Z.W.); Christian.Krautz@uk-erlangen.de (C.K.); Christian.Pilarsky@uk-erlangen.de (C.P.); Robert.Gruetzmann@uk-erlangen.de (R.G.)

**Keywords:** pancreatic cancer, treatment options, surgery, chemotherapy, chemoradiation, therapeutic targets, molecular mechanism, immunotherapy

## Abstract

Pancreatic cancer is one of the most lethal malignancies and is associated with a poor prognosis. Surgery is considered the only potential curative treatment for pancreatic cancer, followed by adjuvant chemotherapy, but surgery is reserved for the minority of patients with non-metastatic resectable tumors. In the future, neoadjuvant treatment strategies based on molecular testing of tumor biopsies may increase the amount of patients becoming eligible for surgery. In the context of non-metastatic disease, patients with resectable or borderline resectable pancreatic carcinoma might benefit from neoadjuvant chemo- or chemoradiotherapy followed by surgeryPatients with locally advanced or (oligo-/poly-)metastatic tumors presenting significant response to (neoadjuvant) chemotherapy should undergo surgery if R0 resection seems to be achievable. New immunotherapeutic strategies to induce potent immune response to the tumors and investigation in molecular mechanisms driving tumorigenesis of pancreatic cancer may provide novel therapeutic opportunities in patients with pancreatic carcinoma and help patient selection for optimal treatment.

## 1. Introduction

**Figure 1:**
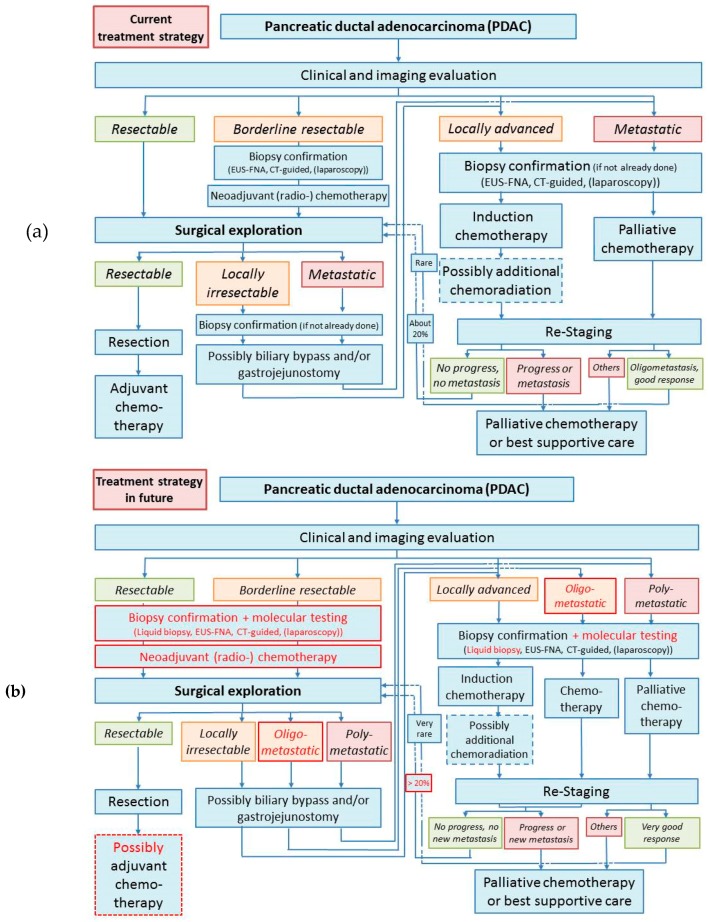
(**a**) Current treatment strategies for pancreatic carcinomas; (**b**) assumed future treatment strategy for pancreatic carcinoma: The most distinctive changes (highlighted in red) are probably (1) an extension of biopsy options and an introduction of routinely molecular tests including chemotherapy sensitivity, (2) an introduction of neoadjuvant therapy even in resectable stage, (3) an improvement of chemotherapy regimens with increased secondary resectability, and (4) the “introduction” of oligometastasis as the fifth subgroup (besides resectable, borderline, locally advanced, and metastatic stages) with enhanced therapy options including surgery in this new group. Dotted frames indicate possible therapy options.

Pancreatic cancer is one of the most lethal malignancies, accounting for the 7th leading cause of cancer-related mortality worldwide [1]. It is estimated that about 458,000 people will be diagnosed with pancreatic cancer worldwide in 2018, and more than 432,000 will die of this disease. 5-year survival in patients with pancreatic cancer is as low as 9% in the USA [2]. Although progress has been made in multimodality treatment with surgery and adjuvant therapy, the mortality rate of pancreatic cancer is still increasing throughout the years. The disappointing prognosis of this disease is largely attributable to its late diagnosis, as most patients with pancreatic cancer remain asymptomatic until the disease develops to an advanced stage [3]. Only non-specific symptoms may exist at early stage, but there are currently no screening programs available [4]. Besides, tumor biology of pancreatic cancer may contribute to its early metastasis. A preclinical study using a mouse model of pancreatic cancer indicates that early metastasis might possibly be detected even when there is no primary tumor found in the pancreas and is associated with epithelial-to-mesenchymal transition and focal inflammation [5]. Therefore, like many other types of cancers, pancreatic cancer is suggested to be a systematic disease, and multidisciplinary management of this disease is of great importance. Treatment of pancreatic cancers includes surgery, chemotherapy, radiation therapy, and palliative care, which are selected on the basis of disease stage (Figure 1). Here we review the current clinical strategies in the treatment of pancreatic ductal adenocarcinoma (PDAC), the most common type of pancreatic cancers, in different scenarios (resectable, borderline resectable, locally advanced, and metastatic PDAC), and potential novel approaches under development based on the expanding molecular biology knowledge.

## 2. Current Management of Pancreatic Cancer

### 2.1. Management of Resectable Pancreatic Cancers

In the management of pancreatic carcinomas, surgical resection is fundamental as surgery is currently the only potential treatment to cure pancreatic cancer that could result in significant improvement in survival, but over 80% of patients with pancreatic cancer are diagnosed when the lesion is no longer primarily resectable [3,6,7]. Therefore, upfront surgery is currently standard in patients with resectable pancreatic carcinomas. The most important goal of surgical resection of pancreatic cancer is to achieve a negative resection margin (R0), as positive resection margin (R1 or R2) is associated with recurrence and dismal prognosis [8,9]. Achieving an R0 margin mandates meticulous perivascular dissection, recognition of the need for vascular resection and reconstruction, as well as potential need for extra-pancreatic organ resection. Additionally, pancreatic cancer surgery is strongly recommended to be performed in specialized high-volume centers, as the hospital volume is reported by many groups associated with resection margin, in-hospital mortality, and long-term survival [10,11,12].

Adjuvant chemotherapy is generally considered, as most patients will develop recurrence after surgical resection. There have been some robust randomized clinical trials evaluating the role of different postoperative chemotherapy regimens, especially for 5-fluorouracil, gemcitabine and combination regimen (e.g., FOLFIRINOX) (Table 1) [13,14,15,16,17,18,19,20,21,22,23,24,25,26,27,28,29,30]. Currently FOLFIRINOX provides the standard adjuvant therapy in patients with high performance status. The effectiveness of postoperative chemoradiotherapy (CRT) remains controversial. The supportive evidence of the use of adjuvant CRT mainly comes from retrospective cohort studies [31,32,33,34]. However, a pooled analysis of nine randomized controlled trials showed that adjuvant CRT results in worse survival than chemotherapy alone [35]. High-quality clinical evidence from randomized controlled trials is limited at present.

Due to lacking evidence, preoperative neoadjuvant treatment could not be recommended for resectable pancreatic cancers but is investigated currently in randomized controlled studies for primary resectable pancreatic carcinoma (Table 2) [30,36,37,38,39,40].

There is currently no reliable clinical evidence or expert consensus to recommend any surveillance strategies after potential curative resection of pancreatic cancer. A retrospective study showed that postoperative surveillance with clinical evaluation and serum carbohydrate antigen (CA) 19-9, together with routine CT and chest X-ray, for every 6 months, is effective and associated with a better survival outcome; however, increased frequency and intensity of surveillance does not bring any benefit [41,42].

For patients experiencing recurrence following resection, the choice of second-line therapies will depend on the site of recurrent disease. Patients with local recurrence will potentially be subjected to surgical resection (if recurrence is in the pancreas or locoregional lymph nodes only) or chemoradiation. Unfortunately, however, most patients with recurrent disease develop metastasis, where systemic chemotherapy may be considered. In rare cases of isolated and limited liver metastasis, resection can improve survival. In addition, palliative and best supportive care is another option, especially for patients with poor performance status.

### 2.2. Management of Borderline Resectable Pancreatic Cancers

In patients with suspected borderline resectable disease, biopsy confirmation of adenocarcinoma with endoscopic ultrasound-guided fine needle aspiration (EUS-FNA), CT-guided biopsy, or staging laparoscopy is required.

After ruling out metastatic disease, neoadjuvant chemotherapy or chemoradiotherapy is applied, preferentially within a clinical trial. In the last years, preoperative neoadjuvant therapy was investigated in order to obtain better local control and eliminate potential micrometastasis of the disease. A recent systematic review of 35 comparative studies demonstrated that neoadjuvant therapy could improve resectability of the disease through down-staging of the tumor, especially in borderline pancreatic cancers [43]. Several large retrospective cohort studies based on a national cancer database also demonstrated that preoperative chemotherapy was associated with improved survival outcome in resected pancreatic cancers of different pathological stages [44,45]. In addition, another systematic review indicated that neoadjuvant therapy is safe and does not affect postoperative complication rates [46]. However, there are currently very few results from randomized trials available with limited quality of evidence (Table 2) [30,36,37,38,39,40]. No clear conclusion could be drawn whether there is advantage of neoadjuvant CRT compared to chemotherapy alone. Many of the larger phase 3 randomized controlled trials are prematurely terminated because of failure of recruitment, as the patients may fear losing the opportunity for surgical resection. Nonetheless, there are still many randomized trials ongoing to explore the potential effects of CRT. For instance, a large phase 3 trial, CONKO-007, was proposed to demonstrate, with 830 participants expected, the effect of CRT as compared to chemotherapy alone in the neoadjuvant setting, which is awaited to disclose meaningful evidence to this field.

After neoadjuvant treatment, surgical exploration is considered with preferable tumor response detected by restaging imaging assessment. Then, resection or palliative operative procedures could be performed depending on the intraoperative findings.

Similar to primary resectable tumors, adjuvant therapies are generally considered as a result of the high risk of recurrence even after radical surgical resection and the association of adjuvant chemotherapy with improved survival in patients with resected pancreatic adenocarcinoma (Table 1) [13,14,15,16,17,18,19,20,21,22,23,24,25,26,27,28,29,30,35,44].

### 2.3. Management of Locally Advanced and Metastatic Pancreatic Cancer

Unresectable pancreatic cancers, both locally advanced and metastatic, might be detected with imaging for staging and resectability assessment or discovered during surgical exploration for patients initially considered as potentially resectable. After biopsy confirmation, the patient’s performance status is evaluated, where patients with good performance status might probably be able to tolerate more aggressive treatment. There have been extensive investigations on various chemotherapeutic regimens, which are basically classified into fluoropyrimidine- and gemcitabine-based regimens (Table 3) [47,48,49,50,51,52,53,54,55,56,57,58,59,60,61,62,63,64,65,66,67,68,69,70,71,72,73,74]. If the patient’s performance status is good (usually determined as Eastern Co-operative of Oncology Group (ECOG) scores 0–1), various combination chemotherapy, including fluoropyrimidine- and gemcitabine-based regimens, are recommended, as a recent Cochrane analysis shows that a combination of several chemotherapeutic agents in advanced pancreatic carcinoma is superior to gemcitabine alone, albeit with a higher side effect profile [75]. Especially the FOLFIRINOX regimen is strongly recommended for patients with locally advanced or metastatic disease. Other combination regimens, especially gemcitabine-based, are usually used for patients with metastatic patients unlikely to tolerate FOLFIRINOX. However, based on the current evidence, it is not possible to determine the optimal gemcitabine combination. The combination of gemcitabine and erlotinib has been associated with significant improvement in overall and progression free survival as compared with gemcitabine alone, although this improvement was small. One characteristic adverse effect of the use of erlotinib is skin rash, and patients who developed skin rash after use of erlotinib might probably have better survival outcomes [56,73]. For patients not fit enough to tolerate combination regimens, gemcitabine monotherapy is considered.

For locally advanced non-metastatic pancreatic cancer, the additional role of radiotherapy has been studied, but the survival benefits of chemoradiotherapy (CRT) are still undetermined. There is currently very limited evidence from randomized clinical trials supporting the use of CRT in this setting. In a systematic review, Huguet and colleagues indicated that CRT increased overall survival when compared with best supportive care or exclusive radiotherapy, but it was more toxic [76]. The advantage of CRT over exclusive chemotherapy is inconsistent (Table 4) [77,78,79,80,81,82,83]. Induction chemotherapy may select patients with locally advanced pancreatic cancer for optimal benefit from CRT by excluding patients with rapid progressive disease. Two retrospective studies indicated significantly improved survival benefit with the use of induction chemotherapy before continuing with CRT, as compared with upfront CRT or with exclusive chemotherapy [84,85]. A recent meta-analysis showed that induction chemotherapy followed by consolidation CRT did not significantly improve survival in patients with locally advanced pancreatic cancer as compared to chemotherapy alone; however, the survival benefit of this treatment strategy over chemotherapy alone was noted only when induction chemotherapy lasted for at least 3 months [86].

In patients with locally advanced unresectable tumor at primary diagnosis, resectability should be reassessed after first treatment. Patients presenting significant response to chemotherapy and/or CRT should undergo surgery if R0 resection seems to be achievable according to the latest imaging as this significantly improves their prognosis (preliminary data from CONKO 007 trial).

When first-line treatment fails, second-line therapy is increasingly considered in patients with good performance status, as it is suggested in association with improved survival [87,88]. There is a paucity of clinical evidence supporting any optimal second-line regimens for patients with advanced pancreatic cancer. For those who have received prior gemcitabine-based therapy, fluoropyrimidine-based chemotherapy regimens are generally accepted as the second-line options. On the other hand, gemcitabine-based therapy could be used to those previously treated with fluoropyrimidine-based therapy. Chemoradiation could be considered in patients with locally advanced disease, but palliative radiotherapy may be administered to patients with locally metastatic disease and poor performance status.

### 2.4. Palliative Care

A considerable proportion of patients with pancreatic cancer require palliative interventions to relieve symptoms and ensure optimal quality of life. Biliary obstruction is one of the most common severe circumstances in patients with pancreatic cancer. Placement of self-expanding metal stents is the preferred method to relieve biliary obstruction in patients with unresectable disease, as it is associated with lower rates of, and longer time to, recurrent biliary obstruction as compared to plastic stents, resulting in less cholangitis [89,90]. Placing the stents endoscopically is preferable as it is safer than percutaneous insertion; however, percutaneous biliary drainage is an alternative option when an endoscopic stent cannot be placed. Another option is surgical biliary bypass, which is considered when the cancer is found unresectable during attempted resection, as it provides durable palliation and potentially avoids additional stent-insertion procedure [91]. For patients with gastric outlet obstruction, endoscopically placed enteral stent is preferred for patients with a short life expectancy or poor performance status, while gastrojejunostomy is considered more effective for patients with longer life expectancy and favorable prognosis [92]. As the quality of life is markedly hampered if gastric outlet obstruction occurs, prophylactic gastrojejunostomy could be considered for those at risk of developing symptomatic gastric outlet obstruction but otherwise fit and with a relatively good prognosis [93].

Most patients with locally advanced or metastatic pancreatic cancer develop cancer-related pain. The mainstay of pain management in these patients is administration of analgesics. However, for those whose analgesic control shows inadequate or with undesirable side effects, EUS- or image-guided celiac plexus neurolysis could significantly improve pain relief [94]. In selected patients with severe local back pain refractory to analgesia, palliative radiation might be considered to ameliorate pain [95]. Except pain, malnutrition is also prevalent in patients with pancreatic cancer. A proper nutritional evaluation should be performed, and oral pancreatic enzyme should be administered in patients with both unresectable and resected pancreatic cancer [96]. Additionally, the risk of developing venous thromboembolism is substantially increased in patients with pancreatic cancer. Low molecular weight heparin is preferably administered, as randomized clinical trials indicated significantly decreased incidence of venous thromboembolism associated with intake of low molecular weight heparins [97,98].

### 2.5. Conclusion/Future Directions

Even with the latest efforts for novel therapeutic strategies, especially new chemo(radio)therapy regimens in (neo)adjuvant settings and improved surgical options, the clinical outcome of patients with pancreatic cancer remains disappointing. Clinically, we anticipate a higher amount of neoadjuvant therapeutic approaches for patients with non-metastatic pancreatic cancer in the near future; however, a better understanding of the underlying molecular mechanisms of this disease is of central importance to design new therapeutic strategies for all patients (Figure 1b). For molecular testing of pancreatic cancer, an individualized therapeutic concept for each patient might be available, thus leading to a better prediction of the patient’s prognosis, a better prediction of the effectiveness of the available chemotherapeutics, and finally improvement in the patient’s outcome.

## 3. Immunotherapy and Other State-of-the-Art Molecular Options

**Figure 2:**
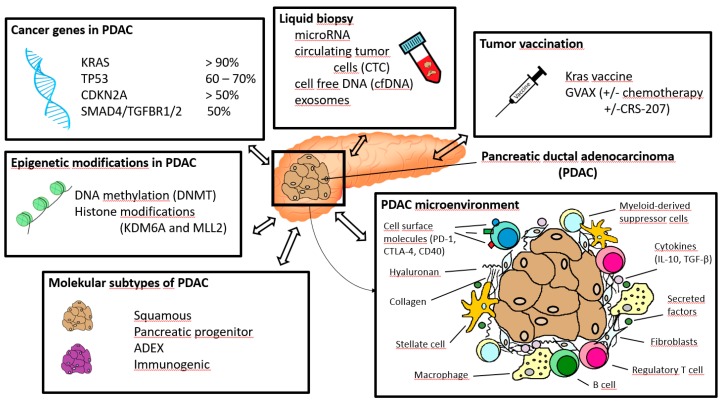
Overview of important aspects in immunotherapy and molecular pathology of pancreatic ductal adenocarcinoma.

Knowledge of the molecular aspects is becoming increasingly important in the therapy and prognosis of patients with ductal pancreatic carcinoma. The understanding of pancreatic carcinoma at the molecular level is a complex interplay of various factors (Figure 2): In patients with pancreatic ductal adenocarcinoma (PDAC) the tumor microenvironment, consisting of cellular and stromal components, plays an important role and influence prognosis leading to development of tumor vaccination as a potential future treatment option. The influence of genes plays a decisive role as well. The most common genes in pancreatic carcinoma are *KRAS*, *TP53*, *CDKN2A,* and *SMAD4/TGFBR1/2*. In addition to the immunological and genetic components, epigenetic modifications and molecular subtypes have a significant influence on PDAC. Promising findings for diagnostic and treatment are expected from liquid biopsy.

### 3.1. Immunotherapy

Pancreatic cancers are characterized by an immunosuppressive microenvironment due to the dysfunction of immune effector CD8+ T cells and Natural killer (NK) cells, which is a result of the involvement of multiple types of immune cells, including cancer-associated fibroblasts, regulatory T cells, myeloid-derived suppressor cells, tumor-associated macrophages, and tumor-infiltrating lymphocytes [99]. The immune suppression occurs through both the expression of tolerance-inducing cell surface molecules (PD-1, CTLA-4, and CD40) and the secretion of immunosuppressing cytokines (IL-10, TGF-β) [100]. The function of the immune system is therefore converted from anti-cancer immunity to a supportive microenvironment that fosters the growth and invasion of the tumor and helps the tumor escape from host immune surveillance. Thus, strategies could be exploited to disrupt this immunosuppressive network and promote the tumoricidal activity of these immune effector cells to potentially improve the outcome of the patients [100,101].

T cells are activated when antigen presenting cells (APCs) present antigen peptides on both major histocompatibility complex (MHC) class I (for CD8+ T cells) and class II (for CD4+ T helper cells) molecules to the T cell receptor (TCR) [100]. Effective activation of T cells also requires additional ligand binding of co-stimulatory receptors, such as CD40, which is a member of the tumor necrosis factor (TNF) receptor family presented on APCs such as tumor-associated macrophages. CD40 on the surface of APCs could bind with the CD40 ligand expressed on activated CD4+ T cells, thus forming a stimulatory loop. CD40 and its ligand (CD154) are both expressed in a subset of pancreatic patients, and the high expression of CD40 ligand is associated with significantly better prognosis than others [102]. The activation of CD40 with a CD40 agonist in combination with gemcitabine is indicated to promote accumulation of tumoricidal macrophages within the tumors in the KPC mouse model, which resulted in stromal collapse and tumor regression in the KPC mouse model and some advanced-stage pancreatic cancer patients [103,104]. On the other hand, inhibitory receptors such as programed cell death-1 (PD-1) and cytotoxic T-lymphocyte associated protein 4 (CTLA-4) expressed on the surface of T cells inhibit T cell activation upon binding to their ligands. PD-1 is an immune checkpoint receptor expressed on activated T cells; whereas PD-1 ligands (PD-L1 and PD-L2) could be expressed on stroma and cancer cells. The binding of PD-1 ligands to PD-1 inactivates T cell responses to the cells presenting these ligands, i.e., the pancreatic cancer cells and stroma in this situation, facilitating the cancer to escape from the host’s immune surveillance [101]. PD-L1 expression, but not PD-L2, is correlated to significantly poorer survival than the ligand-negative patients, and it is inversely correlated with tumor-infiltrating lymphocytes within the tumor, particularly CD8+ T cells [105]. In vivo experiments suggested that blockade of PD-1 signaling with anti-PD-L1 or anti-PD-1 monoclonal antibody promoted CD8+ T cells infiltration into the tumor, induced local immune activation, and finally resulted in substantial anti-tumor effects in a murine pancreatic cancer model [105]. However, the effect of PD-L1 inhibitors in treating patients with pancreatic cancer was low [106]. A preclinical study using the KPC mouse model suggested that depletion of fibroblast-associated protein (FAP)-positive cancer-associated fibroblasts (CAFs) sensitized the tumor to the treatment with anti-PD-L1 monoclonal antibody [107]. FAP-positive CAFs in tumor stroma are the major source of C-X-C motif ligand 12 (CXCL12) in the tumor, and inhibition of its receptor, the C-X-C motif receptor 4 (CXCR4), induced T cell accumulation in the tumor and acted synergistically with anti-PD-L1 in diminishing cancer cells [107]. In addition, PD-L1 inhibitor and gemcitabine chemotherapy also showed synergistic effect on pancreatic cancer in preclinical mouse model [105]. CTLA-4 is another inhibitory checkpoint protein expressed on regulatory T cells and exhausted CD8+ T cells. In a transgenic mouse model, depletion of myofibroblasts induced an immunosuppressive microenvironment, as characterized by decreased overall immune infiltration but increased number of regulatory T cells, which then accelerated the progression of pancreatic cancer leading to reduced survival [108]. The use of anti-CTLA-4 monoclonal antibody reversed the accelerated disease progression and prolonged survival in this CAFs-depleted mouse model. Although single agent Ipilimumab (an anti-CTLA-4 monoclonal antibody) immunotherapy was shown in a phase 2 study ineffective in the treatment of advanced pancreatic cancer, it might be considered when combination chemotherapy with nab-paclitaxel and gemcitabine is applied, as this regimen decreased the number of CAFs in the treatment of pancreatic cancer [109,110].

Tumor vaccination is another immunotherapeutic strategy on the basis that tumor antigens, both mutated proteins present exclusively on cancer cells and normal proteins present at a higher concentration on cancer cells, are expressed by a remarkable proportion of pancreatic tumors [100]. The vaccines could be administered as whole cells, proteins, peptides, DNA, and RNA, which can cause antigen-specific T cell responses that may lead to tumor regression. *KRAS* is one of the most frequent oncogenic mutations present in pancreatic cancers, mostly as a single point mutation at codon 12 resulting in a constitutive activation of *KRAS*, which can be recognized both by T helper cells and cytotoxic T-cells and can be used as an antigen in peptide vaccination for tumors with KRAS mutations [111]. In a phase 1/2 clinical trial, a mutant *KRAS* vaccine, designed to induce T-helper responses, seemed to induce immunologic response in a number of patients with resected pancreatic cancer, and it might be associated with improved long-term immune responses and long-term survival [111]. However, in another study, *KRAS* vaccination for patients with resected pancreatic cancer failed to show elicitable immunogenicity or proven efficacy, although it is safe and tolerable [112]. Whole tumor cell vaccines have the advantage of containing all possible tumor antigens and can be patient-specific. GVAX pancreas is an allogeneic whole-cell pancreatic cancer vaccine generated from pancreatic cancer cell lines modified to express granulocyte-macrophage colony-stimulating factor (GM-CSF) [113]. The rationale for designing the allogeneic GVAX vaccine is that many tumor antigens are commonly expressed among different patients’ cancers, and GM-CSF released by the modified tumor cells induce antigen-presenting cells chemotaxis to the vaccinating tumor, which then phagocytose the tumor cells and present the tumor antigens on MHC class I and II molecules to both CD4+ and CD8+ T cells and thus induce immune-response against the tumor. In several clinical trials, the GVAX pancreas vaccine, in combination with the use of conventional chemotherapy, is shown well-tolerated and induced systemic antitumor immunity to autologous tumor cells in a dose-dependent manner in both resected and advanced pancreatic cancer patients [113,114,115]. Besides, the use of GVAX is seemingly associated with improved survival and correlated with mesothelin-specific CD8+ T cell responses [114,115]. To enhance the efficacy of GVAX pancreas tumor vaccine, cyclophosphamide could be administered to inhibit regulatory T cells [115]. Apart from being used in combination with chemotherapeutic agents, GVAX pancreas could also be administered in combination with CRS-207, a live-attenuated Listeria monocytogenes vaccine expressing mesothelin, which could increase the incidence of mesothelin-specific T cell responses [116]. GVAX/cyclophosphamide in combination with CRS-207 for second-line treatment was suggested to extend survival in patients with metastatic pancreatic cancer [117]. In addition, the combination of GVAX and immune checkpoint inhibitors might provide further benefit to patients with pancreatic cancer and could be evaluated in clinical trials [118,119]. Nevertheless, the use of a telomerase peptide vaccine, GV-1001, in combination with modern chemotherapeutic agents gemcitabine and capecitabine did not show any survival advantage when compared to chemotherapy alone in patients with locally advanced or metastatic pancreatic cancers [120].

Other immunotherapeutic strategies have also been tested in patients with pancreatic cancers; however, most of them failed to show any clinically meaningful effects as had been found in other malignancies. Therefore, new strategies of immunotherapy to induce potent immune response to the tumors need to be developed. Investigation in molecular mechanisms that underlie the tumorigenesis of pancreatic cancer may provide novel therapeutic opportunities in these patients and instruct patient selection for optimal treatment.

### 3.2. Molecular Pathology of Pancreatic Ductal Adenocarcinoma

Cancer is a genetic disease caused by the accumulation of somatic mutations in oncogenes and tumor suppressor genes. Genetic analyses reveal four major oncogenes (*KRAS*, *CDKN2A*, *TP53*, *SMAD4*), amongst other mutated genes present at lower prevalence, involved in the development of pancreatic ductal adenocarcinoma [121]. Somatic activating mutations of the *KRAS* oncogene, which encodes a ~21 kDa small GTPase, are present in over 90% of PDAC patients [122]. In PDACs, the activating mutations of *KRAS* are mostly point mutations at codon 12, leading to constitutive activation of KRAS protein and persistent stimulation of downstream pathways. Three major *KRAS* downstream pathways are identified in PDAC, including Raf/Mek/Erk, PI3K/Pdk1/Akt, and the Ral guanine nucleotide exchange factor pathway. In vivo analyses with mouse models demonstrate that sustained oncogenic *KRAS* signaling is essential for both the progression and maintenance of PDAC and the growth and maintenance of its metastatic lesions [123,124,125]. Apart from advanced PDAC, somatic *KRAS* activating mutation is also present in most low-grade pancreatic intraepithelial neoplasms (PanINs)—the most common precursor lesion of PDAC, indicating that *KRAS* mutation is one of the earliest alterations in the initiation of pancreatic tumorigenesis [126]. However, low frequency of spontaneous progression of precursor lesions to invasive PDAC suggests that additional genetic aberrations (*CDKN2A*, *TP53*, or *SMAD4*) are needed for disease progression [127].

Three major tumor suppressor genes (*CDKN2A*, *TP53*, and *SMAD4*) have been identified frequently mutated in PDAC, and they are strongly associated with malignant behavior of the tumor and may predict poor survival in patients with resectable pancreatic cancers [128]. The cyclin dependent kinase inhibitor 2A (*CDKN2A*), which functions in cell cycle control by a combination of 2 CDK kinase inhibitor isoforms and 1 alternate open reading frame isoform as p53 protein stabilizer, is the most frequent altered tumor suppressor gene, with loss-of function mutations in more than 90% of PDAC [129,130]. Alterations in *CDKN2A* are also early events, with loss of protein function in a subset of low-grade PanINs [126,131]. On the contrary, alterations of *TP53* and *SMAD4* expression are late events, occurring only in high grade PanINs or invasive carcinomas [131,132]. Somatic mutations in the tumor suppressor gene TP53, which responds to diverse cellular stresses by inducing cell cycle arrest and apoptosis, are also frequently shown in PDACs [133]. In addition to pancreatic cancers, mutations in this gene are also associated with a wide range of human cancers. Somatic inactivation of tumor suppressor gene *SMAD* family member 4 (*SMAD4*) occurs in greater than 50% of PDACs [134]. *SMAD4* encodes a signal transduction protein downstream of the transforming growth factor β (TGF-β) pathway. In response to the activating signaling from TGF-β, Smad 2/3, the TGF-β receptor substrates, are phosphorylated and associated with Smad 4, forming a Smad complex, which then translocates into the nucleus and regulates gene expression by interacting with DNA and DNA-binding proteins [134]. In normal pancreas cells, the TGF-β/SMAD4 signaling pathway induces a tumor suppressive effect by activation of cell cycle arrest, apoptosis of epithelial cells, and the maintenance of genomic integrity; whereas, in PDACs, due to inactivation or loss of SMAD4, SMAD4-independent TGF-β signaling pathways are triggered, resulting in reduced cell cycle arrest and apoptosis, promotion of epithelial-to-mesenchymal transition and angiogenesis and induction of immune suppression, all of which lead to progression and metastasis of cancer cells [134]. However, studies using genetically engineered mice show that pancreas-specific *SMAD4* deficiency does not initiate either PanIN or invasive PDAC; *SMAD4* loss markedly promotes tumor progression initiated by *KRASG12D* activating mutation, indicating that blockade of TGF-β signaling and activation of Ras signaling cooperate to promote PDAC progression [135,136]. In addition, overexpression of TGF-β activates Ras/Erk, P13K/AKt, p38 MAPK, and NF-κB pathways, all of which play a role in PDAC tumorigenesis.

Apart from these well-characterized oncogene and tumor suppressor genes, other genes are altered at lower prevalence in PDACs but may still play a pivotal role in pancreatic oncogenesis. For instance, *GPRC5A*, a member of the G protein-coupled receptor family, is shown upregulated in pancreatic cancer primary and metastatic lesions as compared with normal tissues, and it promotes the growth and migration of pancreatic cancer cells [137]. The standard chemotherapy treatment with gemcitabine increased the expression of *GPRC5A* by the interaction between its mRNA and RNA-binding protein HuR; whereas, knockout of *GPRC5A* sensitized pancreatic cancers to gemcitabine chemotherapy. Knockout of *GPRC5A* reduced the proliferation and migration ability of PDAC cells and suppressed chemotherapy resistance with various chemotherapy agents currently in clinical use, suggesting that targeting *GPRC5A* may have the potential to improve chemotherapy efficacy [138]. The RIP 1/3 proteins, the main components of the necrosome, are also shown highly expressed in PDAC and are pivotal in the process of necroptosis, another type of programed cell death [139]. The RIP 1/3 promote PDAC oncogenesis and induce the immunosuppressive tumor microenvironment through both the necroptosis-induced CXCL1 and Mincle signaling; whereas, depletion of RIP 1/3, CXCL1, or Mincle protected against PDAC progression and restored anti-tumor immunity in vivo.

Moreover *BRCA1* and *BRCA2* mutations resulting in DNA damage repair deficiency and increasing especially the risk for breast and ovarian cancer are the most common causes of familial pancreatic cancer. In familial pancreatic cancer, *BRCA2* is mutated in about 5% to 10% of cases and *BRCA1* in approximately 1% [140].

#### 3.2.1. Molecular Subtypes of Pancreatic Cancers and Other Considerations Based on Genomic and Transcriptomic Analyses

Whole-exome and genome sequencing has revealed that human PDAC is an extremely heterogeneous disease with diverse molecular subtypes [141,142,143,144,145,146,147]. Although the major oncogenic mutations are pivotal in the tumorigenesis of PDAC, most other genes are mutated in only a small proportion of tumors. These genes, however, are typically a part of or affect several common signaling pathways. Studies with these cutting-edge analyses show that there are several principal signaling pathways that are genetically altered in most PDACs, but specific genes altered in any individual tumor are largely different [141,142]. This molecular heterogeneity of PDACs may explain, at least in part, the reason why agents targeting specific oncogenic genes have mostly failed to benefit unselected patient populations. Thus, drugs targeting a signaling pathway or a key point of the pathway could potentially improve treatment outcomes and warrant further development.

Many studies set out to define molecular subtypes of PDACs based on the genetic mutational characteristics of the tumor to provide evidence for patient selection for optimal treatment [144,145,146,147]. In the most recent study conducted by an international research group, Bailey and colleagues, with the use of transcriptomic sequencing, propose 4 molecular subtypes of PDACs: squamous, pancreatic progenitor, aberrantly differentiated endocrine exocrine (ADEX), and immunogenic based on the differential expression of transcription factors and downstream targets [147]. Squamous tumors are characterized by the alteration of gene networks involved in inflammation, hypoxia response, metabolic reprogramming, TGF-β signaling, and autophagy. They are also associated with mutations in *TP53* and *KDM6A*, activation of MYC signaling, α6β1 and α6β4 integrin signaling, and EGF signaling pathways, upregulation of TP63ΔN network, as well as hypermethylation of pancreatic endodermal cell-fate determining genes (PDX1, MNX1, GATA6, and HNF1B). In addition, pancreatic squamous tumors have a poor prognosis. Pancreatic progenitor tumors are defined by the preferential expression of genes involved in pancreatic endoderm cell-fate determination towards a pancreatic lineage that are pivotal for early pancreatic development, including transcription factors PDX1, MNX1, HNF4G, HNF4A, HNF1B, HNF1A, FOXA2, FOXA3, and HES1. Besides, alterations in genes regulating fatty acid oxidation, steroid hormone biosynthesis, drug metabolism and O-linked glycosylation of mucins, as well as TGFBR2 inactivation are also typical in this subtype. The ADEX class, which is a subclass of the pancreatic progenitor tumors, displays upregulation of transcriptional networks involved in later stages of pancreatic development and differentiation. Transcriptional networks involved in both exocrine (NR5A2, MIST1, RBPJL and their downstream targets) and endocrine (INS, NEUROD1, NKX2-2, and MAFA) differentiation at later stages are upregulated, rather than one or the other as is the case in normal pancreas development. The immunogenic subtype, apart from sharing many molecular characteristics with pancreatic progenitor tumors, is associated with upregulated immune networks including B cell signaling pathways, antigen presentation, CD4+ T cell, CD8+ T cell and Toll-like receptor signaling pathways. Additionally, the study also identified 32 recurrently mutated genes aggregating into 10 signaling pathways, including KRAS, TGF-β, WNT, NOTCH, ROBO/SLIT signalling, G1/S transition, SWI-SNF, chromatin modification, DNA repair and RNA processing. The identification of signaling pathways important in PDAC tumorigenesis and molecular classification provides the most pivotal insights in improving the clinical outcomes of PDAC patients by selecting patients for the optimal therapy.

Recent studies based on novel genomic analysis and informatics methods also challenge the conventional notion of pancreatic cancer progression model [148]. PDAC tumorigenesis may neither be gradual nor follow the currently prevailing model of gene alteration sequence (*KRAS*, followed by *CDKN2A*, then *TP53* and *SMAD4*). On the contrary, simultaneous gene rearrangements associated with mitotic errors occur at early stages of tumorigenesis and confer its invasive and metastatic properties, which is supported by the observation that early stage PanIN2 lesions share a great proportion of somatic mutations required for PDAC development with the invasive cancer [149].

#### 3.2.2. Epigenetic Modifications in the Tumorigenesis of PDACs

Another concern in the initiation and progression of PDAC is the epigenetic modifications of oncogenes and tumor suppressor genes, which alter the conformation of the chromatin and histones in a reversible manner, leading to changes in gene promoter accessibility and gene expression [150]. The best characterized mechanisms of epigenetic regulations that play a role in the tumorigenesis of PDACs include DNA methylation and histone modifications (such as methylation and acetylation). DNA methylation of tumor suppressor genes *APC*, *BRCA1*, *CDKN2A* at their promoter regions blocks transcription activity, which is thought to be associated with human PDACs [150]. Genome-wide DNA methylation analysis in PDACs shows that aberrant hypermethylation is prevalent at 5’ regions with reduced mRNA expression levels and is involved in key molecular mechanisms important to PDAC, including TGF-β, Wnt, integrin signaling, cell adhesion, stellate cell activation, and axon guidance signaling pathways [151]. DNA methylation is carried out by 3 active DNA methyltransferases (DNMTs), including DNMT1, DNMT3A, and DNMT3B, which transfer a methyl group from S-adenosylmethionine to cytosine residues in DNA. All 3 DNMTs have been shown increased in PDACs and are associated with lower overall survival [152,153]. Several DNMT inhibitors have been developed and shown some promising results, but their efficacy in the treatment of pancreatic cancer still needs further investigations [150].

Histone methylation and acetylation are the 2 most important types of histone modifications associated with pancreatic tumorigenesis. Histone methylation is regulated by histone methyltransferases (HMTs) and histone demethylases (HDMs) in a reversible manner, mostly on the lysine residues of histone amino acid side chains [150]. The effect of histone methylation varies depending on the position of methylated lysine residue and the extent of methylation (mono-, di, or tri-methylation). A recent whole genome sequencing and copy number analysis identified KDM6A and MLL2 as the most frequently altered histone methylation regulatory genes in pancreatic cancers [146]. KDM6A is an H3K27me3 demethylase, which is also crucial for pancreas endodermal differentiation through regulating Wnt signaling pathway [154]. Enrichment of H3K27me3 marks on gene promoter regions is associated with suppressed expression of the genes. H3K27 methylation is carried out by the histone methyltransferase EZH2, a component of the polycomb group protein complex, which is upregulated within the cell nucleus in PDAC and possibly contributes to its progression [155]. However, a study with pancreatic tissue sample indicates that H3K27me3 expression is significantly reduced in pancreatic cancer as compared to normal tissues and is associated with worse overall survival [156]. On the other hand, MLL2 is a methyltransferase for H3K4 (an activating epigenetic mark) and is shown altered in a smaller proportion of PDACs [156]. Histone acetylation is the most well-studied histone modification. The acetylation of histones, with an acetyl group attached to the lysine residues of histone N-terminal tails, generally results in relaxed chromatin conformation and transcription activation [150]. It is a balanced dynamic process controlled by the reverse regulatory functions of histone acetyltransferases (HATs) and histone deacetylases (HDACs); whereas, aberrant balance of HATs and HDACs is implicated in a variety of human diseases, including pancreatic cancers. HDACs 1, 2, 3, and 7 have been found overexpressed in a subset of PDACs, which all play an important role in the development of the disease [157]. However, the functions of HATs have been far less investigated. The HAT p300 seems to possess multifaceted functions in the tumorigenesis of PDACs: it shows decreased expression in highly metastatic PDAC cell lines, suggesting a metastasis-suppressive role in PDACs; and it is also involved in the activation of c-myc gene, suggesting its growth-promoting function in PDACs [157]. Various agents targeting HATs and HDACs have been intensively tested in preclinical and clinical studies, but their effects are generally disappointing [158]. Hence, novel agents with highly selective targeting functions and the underlying molecular mechanisms of epigenetic modifications clearly warrant further exploration.

Additionally, posttranscriptional RNA modification is another aspect of epigenetic regulation. N6-methyladenosine (m6A) is the most abundant mRNA modification in mammals, which is a reversible process regulated by a group of RNA methyltransferases and demethylases, regulating RNA stabilities, mRNA splicing, microRNA processing, and mRNA translation. A recent preclinical study shows that methyltransferase-like 3 (METTL3), an RNA m6A methyltransferase, is involved in the chemo- and radioresistance in pancreatic cancer cells, although it does not show any effect on the morphology and proliferation of the cells [159]. On the other hand, another study shows that ALKBH5, an RNA demethylase, is down-regulated in pancreatic cancer cells, and is associated with the significant enrichment of total RNA methylation in PDAC cells [160]. The cellular and molecular processes of RNA methylation underlying PDAC development still needs further investigation.

Two further proteins playing an important role in epigenetic regulation of PDAC are UNR (Upstream of N-ras) and PIWI (P-element-induced wimpy testis). Low UNR expression was significantly associated with shorter progression-free survival after surgery [161]. Human P-element-induced wimpy testis 1 and 2 (PIWIL1 and PIWIL2) proteins act as protectors of germline and correlate with factors associated to the progenitor molecular subtype of PDAC [162]. Both proteins could be a potential prognostic biomarker for resectable PDAC, improving subsequent adjuvant treatment decisions.

## Figures and Tables

**Table 1:** Summary of randomized controlled trials concerning adjuvant chemotherapy in patients with pancreatic carcinomas.

Trial	Year	Country/Region	N	Regimens	Survival Outcomes
Bakkevold [13]	1993	Norway	47	AMF (5-FU, doxorubicin, mitomycin C) (*n* = 23) vs. observation (*n* = 24)	mOS 23 mo vs. 11 mo (*p* = 0.02); 2-year survival 43% vs. 32%; 5-year survival 4% vs. 8%
Takada [14]	2002	Japan	158	MF (5-FU and mitomycin C) (*n* = 81) vs. observation (*n* = 77)	5-year OS 17.8% vs. 26.6% in patients with curative resection (*p* = 0.4544); 5-year DFS 8.6% vs. 7.8% (*p* = 0.8372)
ESPAC-1 [15]	2004	Europe	289	5-FU/folinic acid (with and without chemoradiotherapy) (*n* = 147) vs. no chemotherapy (observation and chemoradiotherapy) (*n* = 142)	mOS 20.1 mo vs. 15.5 mo (*p* = 0.009); 2-year estimated OS 40% vs. 30%; 5-year estimated OS 21% vs. 8%; mDFS 15.3 mo vs. 9.4 mo (*p* = 0.02); 1-year DFS 58% vs. 43%
JSAP [16]	2006	Japan	89	5-FU/cisplatin (*n* = 45) vs. observation (*n* = 44)	mOS 12.5 mo vs. 15.8 mo; 5-year OS 26.4% vs. 14.9%
CONKO-001 [17,18]	2007 and 2013	Germany and Austria	368	Gemcitabine 6 cycles (*n* = 179) vs. observation (*n* = 175)	mOS 22.8 mo vs. 20.2 mo (*p* = 0.01); 5-year OS 20.7% vs. 10.4%; 10-year OS 12.2% vs. 7.7%; mDFS 13.4 mo vs. 6.9 mo (*p <* 0.001); 3-year DFS 23.5% vs. 7.5%; 5-year DFS 16.5% vs. 5.5%
Yoshitomi [19]	2008	Japan	100	Gemcitabine and UFT (uracil/tegafur) (*n* = 50) vs. gemcitabine (*n* = 49)	mOS 21.2 mo vs. 29.8mo (*p* = 0.28); 1-year OS 80.0% vs. 85.7%; 3-year OS 30.4% vs. 46.9%; mDFS 12.3 mo vs. 12.0 mo (*p* = 0.67); 1-year DFS 50.0% vs. 49.0%; 3-year DFS 17.7% vs. 21.6%
ESPAC-1 plus [20]	2009	Europe	192	5-FU/folinic acid (*n* = 97) vs. observation (*n* = 95)	mOS 24.0 mo vs. 12.8 mo; 2-year OS 49% vs. 28%; 5-year OS 24% vs. 14%
ESPAC-3 v1 [20]	2009	Europe	122	5-FU/folinic acid (*n* = 61) vs. observation (*n* = 61)	mOS 25.9 mo vs. 20.3 mo; 2-year OS 54% vs. 48%; 5-year OS 20% vs. 20%
JSAP-02 [21]	2009	Japan	119	Gemcitabine 3 cycles (*n* = 58) vs. observation (*n* = 60)	mOS 22.3 mo vs. 18.4 mo (*p* = 0.19); mDFS 11.4 mo vs. 5.0 mo (*p* = 0.01)
ESPAC-3 v2 [22]	2010	International	1088	5-FU/folinic acid (*n* = 551) vs. gemcitabine (*n* = 537) for 6 mo	mOS 23.0 mo vs. 23.6 mo (*p* = 0.39); estimated 2-year OS 48.1% vs. 49.1%; mPFS 14.1 mo vs. 14.3 mo (*p* = 0.53); estimated 2-year PFS 30.7% vs. 29.6%
RTOG 97-04 [23,24]	2011	USA and Canada	451	5-FU (*n* = 230) vs. gemcitabine (*n* = 221), both with (before and after) CRT (5-FU and 50.4 Gy)	For pancreatic head tumors, mOS 17.1 mo vs. 20.5 mo (*p* = 0.12); 5-year OS 18% vs. 22%
PACT-7 [25]	2012	Italy and Switzerland	102	Gemcitabine (*n* = 51) vs. PEFG (cisplatin, epirubicin, 5-FU, gemcitabine) (*n* = 49), both followed by chemoradiation (5-FU and 54–60 Gy)	mOS 24.8 mo vs. 28.9 mo; mDFS 11.7 mo vs. 15.2 mo; 1-year DFS 49.0% vs. 69.4%
Shimoda [26]	2015	Japan	57	S-1 (*n* = 29) vs. gemcitabine (*n* = 28)	mOS 21.5 mo vs. 18.0 mo (*p* = 0.293); 2-year OS 46% vs. 38%; mDFS 14.6 mo vs. 10.5 mo (*p* = 0.188); 2-year DFS 41% vs. 18%
JASPAC 01 [27]	2016	Japan	385	S-1 (*n* = 187) vs. gemcitabine (*n* = 190)	mOS 46.5 mo vs. 25.5 mo (*p <* 0.0001); 5-year OS 44.1% vs. 24.4%; mRFS 22.9 mo vs. 11.3 mo (*p <* 0.0001); 5-year RFS 33.3% vs. 16.8%; recurrence 66% vs. 78%
ESPAC-4 [28]	2017	Europe	732	Gemcitabine and capecitabine (*n* = 364) vs. gemcitabine (*n* = 366)	mOS 28.0 mo vs. 25.5 mo (*p* = 0.032); estimated 1-year OS 84.1% vs. 80.5%; estimated 2-year OS 53.8% vs. 52.1%; in R1 patients, mOS 23.7 mo vs. 23.0 mo; in R0 patients, mOS 39.5 mo vs. 27.9 mo (*p* = 0.0001)
CONKO-005 [29]	2017	Germany	436	Gemcitabine and erlotinib (*n* = 219) vs. gemcitabine (*n* = 217)	mOS 24.5 mo vs. 26.5 mo (*p* = 0.61); estimated 2-year OS 53% vs. 54%; estimated 5-year OS 23% vs. 20%; mDFS 11.4 mo vs. 11.4 mo (*p* = 0.26); estimated 2-year DFS 25% vs. 25%; estimated 5-year DFS 12% vs. 11%
PACT-15 [30]	2018	Italy	93	Gemcitabine 6 cycles (*n* = 26) vs. PEXG (gemcitabine, cisplatin, epirubicin, capecitabine) 6 cycles (*n* = 30)	mOS 20.4 mo vs. 26.4 mo; 3-year OS 35% vs. 43%; 5-year OS 13% vs. 24%; mDFS 4.7 mo vs. 12.4 mo; 1-year DFS 23% vs. 50%

**Table 2:** Summary of randomized controlled trials concerning neoadjuvant chemo(radio-)therapy in patients with pancreatic carcinomas.

Trial	Year	Country/Region	N	Regimens	Outcomes
**Neoadjuvant chemotherapy**					
Palmer [36]	2007	UK	50, resectable PC	Gemcitabine (*n* = 24) vs. gemcitabine and cisplatin (*n* = 26)	Resection rate 38% vs. 70%; R0 resection 75% vs. 75%; mOS 9.9 mo vs. 15.6 mo; 1-year OS 41.7% vs. 61.5%
Sahora [37]	2014	Austria	30, 11x borderline resectable and 19x locally advanced	Gemcitabine 4 cycles and bevacizumab 3 doses (*n* = 11) vs. gemcitabine 4 cycles and bevacizumab 6 doses (*n* = 19)	resection rate 36.4% vs. 36.8% (*p* = 0.97)
PACT-15 [30]	2018	Italy	93	PEXG (gemcitabine, cisplatin, epirubicin, capecitabine) 3 cycles before and after surgery (*n* = 29) vs. PEXG 6 cycles after surgery	mOS 38.2 mo vs. 26.4 mo; 3-year OS 55% vs. 43%; 5-year OS 49% vs. 24%; mDFS 16.9 mo vs. 12.4 mo; 1-year DFS 66% vs. 50%
**Neoadjuvant CRT**					
E1200 [38]	2010	USA	23	CRT (gemcitabine and 50.4Gy) (*n* = 10) vs. chemotherapy (gemcitabine, cisplatin, 5-FU) followed by CRT (5-FU, 50.4Gy) (*n* = 11), both followed by surgery and gemcitabine adjuvant chemotherapy	mOS 19.4 mo vs. 13.4 mo; 1-year acturial OS 69% vs. 61%; 2-year acturial OS 32% vs. 13%; mPFS 14.2 mo vs. not given; 1-year acturial PFS 59% vs. 15%; resectability 30% vs. 18.2%
Golcher [39]	2015	Germany and Switzerland	73	Upfront surgery (*n* = 33) vs. neoadjuvant CRT (gemcitabine, cisplatin, and 55.8 Gy) followed by surgery (*n* = 33), both followed by gemcitabine chemotherapy	mOS 14.4 mo vs. 17.4 mo (*p* = 0.96); mPFS 8.7 mo vs. 8.4 mo (*p* = 0.95); R0 rate 48% vs. 52% (*p* = 0.81); pN0 rate 30% vs. 39% (*p* = 0.44)
Casadei [40]	2015	Italy	38	Upfront surgery (*n* = 20) vs. neoadjuvant CRT (gemcitabine) followed by surgery (*n* = 18), both followed by gemcitabine chemotherapy	mOS 19.5 mo vs. 22.4 mo (*p* = 0.973); resectability 75% vs. 61.1% (*p* = 0.489); R0 rate 25.0% vs. 38.9% (*p* = 0.489)

**Table 3:** Summary of randomized controlled trials concerning first-line chemotherapy in patients with locally advanced or metastatic pancreatic carcinomas.

Trial	Year	Country/Region	N	Regimens	Outcomes
Burris [47]	1997	USA and Canada	126	Gemcitabine (*n* = 63) vs. 5-FU (*n* = 63)	mOS 5.65 mo vs. 4.41 mo (*p* = 0.0025); 1-year OS 18% vs. 2%; mPFS 2.33 mo vs. 0.92 mo (*p* = 0.0002); 1-year PFS 9% vs. 5%; clinical benefit response 23.8% vs. 4.8 (*p* = 0.0022)
Huguier [48]	2001	France	45	5-FU + leucovorin + cisplatin (*n* = 22) vs. best supportive care (*n* = 23)	mOS 8.6 mo vs. 7.0 mo
Ducreux [49]	2002	France	207	5-FU + cisplatin (*n* = 104) vs. 5-FU (*n* = 103)	response 12% vs. 0% (*p <* 0.01); 1-year OS 17% vs. 9% (*p* = 0.10); 1-year PFS 10% vs. 0% (*p* = 0.0001)
Colucci [50]	2002	Italy	107	Gemcitabine + cisplatin (*n* = 53) vs. gemcitabine (*n* = 54)	mOS 30 weeks vs. 20 weeks (*p* = 0.43); response 26.4% vs. 9.2% (*p* = 0.02); mTTP (time to progression) 20 weeks vs. 8 weeks (*p* = 0.048)
Scheithauer [51]	2003	Austria	83	Gemcitabine + capecitabine (2500 mg/m2 qd 1/2 weeks) (*n* = 41) vs. gemcitabine (high-dose intense) (*n* = 42)	mOS 9.5 mo vs. 8.2 mo; 1-year OS 31.8% vs. 37.2%; mPFS 5.1 mo vs. 4.0 mo; response 17% vs. 14%; clinical benefit 48.4% vs. 33%; P values not reported
Tempero [52]	2003	USA and Netherlands	92	dose-intense gemcitabine (*n* = 49) vs. fixed dose rate gemcitabine (*n* = 43)	mOS 5.0 mo vs. 8.0 mo (*p* = 0.013); 1-year OS 9% vs. 28.8% (*p* = 0.014); 2-year OS 2.2% vs. 18.3% (*p* = 0.007); mPFS 1.9 mo vs. 3.4 mo (*p* = 0.68) mTTF (time to treatment failure) 1.8 mo vs. 2.1 mo (*p* = 0.09)
Ducreux [53]	2004	France	63	5-FU + oxaliplatin (*n* = 31) vs. 5-FU (*n* = 15) vs. oxaliplatin (*n* = 17)	mOS 9.0 mo vs. 2.4 mo vs. 3.4 mo; mPFS 4.2 mo vs. 1.5 mo vs. 2.0 mo; response 10% vs. 0% vs. 0%; stable 48% vs. 20% vs. 12%
Louvet (GERCOR GISCAD) [54]	2005	France and Italy	326	Gemcitabine + oxaliplatin (*n* = 157) vs. gemcitabine (*n* = 156)	mOS 9.0 mo vs. 7.1 mo (*p* = 0.13); 1-year OS 34.7% vs. 27.8% (*p* = 0.22); mPFS 5.8 mo vs. 3.7 mo (*p* = 0.04); response 26.8% vs. 17.3% (*p* = 0.04); clinical benefit 38.2% vs. 26.9% (*p* = 0.03)
Heinemann [55]	2006	Germany	195	Gemcitabine + cisplatin (*n* = 98) vs. gemcitabine (*n* = 97)	mOS 7.5 mo vs. 6.0 mo (*p* = 0.15); 1-year OS 25.3% vs. 24.7% (*p* = 0.21); mPFS 5.3 mo vs. 3.1 mo (*p* = 0.053); response 10.2% vs. 8.2% ns; stable 60.2% vs. 40.2% (*p <* 0.001)
Moore (NCIC CTG PA.3) [56]	2007	International	569	Gemcitabine plus erlotinib (*n* = 285) vs. gemcitabine plus placebo (*n* = 284)	mOS 6.24 mo vs. 5.91 mo (*p* = 0.038); 1-year OS 23% vs. 17% (*p* = 0.023); mPFS 3.75 mo vs. 3.55 mo (*p* = 0.004); control 57.5% vs. 49.2% (*p* = 0.07)
Herrmann [57] (SAKK 44/00-CECOG/PAN.1.3.001)	2007	Europe	319	Gemcitabine + capecitabine (650 mg/m2 bid po 2/3 weeks) (*n* = 160) vs. gemcitabine (standard dose) (*n* = 159)	mOS 8.4 mo vs. 7.2 mo (*p* = 0.234); 1-year OS 32% vs. 30%; mPFS 4.3 mo vs. 3.9 mo (*p* = 0.103); response 10.0% vs. 7.8%; clinical benefit 19% vs. 20%
Boeck [58]	2008	Germany	190	Capecitabine plus oxaliplatin (*n* = 61) vs. capecitabine plus gemcitabine (*n* = 64) vs. gemcitabine plus oxaliplatin (*n* = 63)	mOS 8.1 mo vs. 9.0 mo vs. 6.9 mo (*p* = 0.56); 1-year OS 29% vs. 33% vs. 22%; mPFS 4.2 mo vs. 5.7 mo vs. 3.9 mo (*p* = 0.67); 1-year PFS 8% vs. 14% vs. 8%; response 13% vs. 25% vs. 13% (*p* = 0.13)
Cunningham [59]	2009	UK	533	Gemcitabine + capecitabine (830 mg/m2 bid po 3/4 weeks) (*n* = 267) vs. gemcitabine (standard dose) (*n* = 266)	mOS 7.1 mo vs. 6.2 mo (*p* = 0.08); 1-year OS 24.3% vs. 22.0%; mPFS 5.3 mo vs. 3.8 mo (*p* = 0.004); 1-year PFS 13.9% vs. 8.4%; response 19.1% vs. 12.4% (*p* = 0.03)
Poplin (E6201) [60]	2009	USA	824	Gemcitabine (*n* = 275) vs. fixed dose rate gemcitabine (*n* = 277) vs. gemcitabine plus oxaliplatin (*n* = 272)	mOS 4.9 mo vs. 6.2 mo vs. 5.7 mo; 1-year OS 16% vs. 22% vs. 21%; 2-year OS 4% vs. 6% vs. 6%; mPFS 2.6 mo vs. 3.5 mo vs. 2.7 mo
Kulke (CALGB 89904) [61]	2009	USA	245	Gemcitabine plus cisplatin (*n* = 62) vs. fixed dose rate gemcitabine (*n* = 58) vs. gemcitabine plus docetaxel (*n* = 65) vs. gemcitabine plus irinotecan (*n* = 60)	mOS 6.7 mo vs. 6.4 mo vs. 6.4 mo vs. 7.1 mo; mTTP 4.5 mo vs. 3.3 mo vs. 4.1 mo vs. 4.0 mo; response 13% vs. 14% vs. 12% vs. 14%
Colucci (GIP-1) [62]	2010	Italy	400	Gemcitabine + cisplatin (*n* = 201) vs. gemcitabine (*n* = 199)	mOS 7.2 mo vs. 8.3 mo (*p* = 0.38); 1-year OS 30.7% vs. 34.0%; mPFS 3.8 mo vs. 3.9 mo (*p* = 0.80); 1-year PFS 14.5% vs. 12.8%; response 12.9% vs. 10.1% (*p* = 0.37); clinical benefit 15.1% vs. 23.0% (*p* = 0.057)
Dahan (FFCD 0301) [63]	2010	France	202	5-FU/folinic acid/cisplatin followed by gemcitabine (*n* = 102) vs. gemcitabine followed by 5-FU/folinic acid/cisplatin (*n* = 100)	mOS 6.7 mo vs. 8.03 mo (*p* = 0.83); mPFS 3.4 mo vs. 3.5 mo (*p* = 0.67); response 15% vs. 19%
PRODIGE 4/ ACCORD 11 [64]	2011	France	342	FOLFIRINOX (*n* = 171) vs. gemcitabine (*n* = 171)	mOS 11.1 mo vs. 6.8 mo (P <0.001); 1-year OS 48.4% vs. 20.6%; mPFS 6.4 mo vs. 3.3 mo (*p <* 0.001); 1-year PFS 12.1% vs. 3.5%; response 31.6% vs. 9.4% (*p <* 0.001); 6-month degradation QoL 31% vs. 66% (*p <* 0.001)
Ozaka (JACCRO PC-01) [65]	2012	Japan	112	Gemcitabine plus S-1 (*n* = 53) vs. gemcitabine (*n* = 59)	mOS 13.7 mo vs. 8.0 mo (*p* = 0.035); 1-year OS 55.9% vs. 29.0%; mPFS 6.15 mo vs. 3.78 mo (*p* = 0.0007); response 28.3% vs. 6.8% (*p* = 0.005)
Nakai (GEMSAP) [66]	2012	Japan	106	Gemcitabine plus S-1 (*n* = 53) vs. gemcitabine (*n* = 53)	mOS 13.5 mo vs. 8.8 mo (*p* = 0.104); 1-year OS 52.8% vs. 30.2% (*p* = 0.031); mPFS 5.4 mo vs. 3.6 mo (*p* = 0.036); response 18.9% vs. 9.4% (*p* = 0.265)
Chao [67]	2013	Taiwan	46	Gemcitabine + ciaplatin (*n* = 21) vs. gemcitabine (*n* = 25)	mOS 7.9 mo vs. 7.7 mo (*p* = 0.752); 1-year OS 9.5% vs. 12%; mTTP 3.6 mo vs. 4.6 mo (*p* = 0.857); partial response 4.8% vs. 8% (*p* = 1); clinical benefit 29% vs. 36% (*p* = 0.592)
Von Hoff and Goldstein (MPACT) [68]	2013 and 2015	International 11 countries	861	Gemcitabine + nab-paclitaxel (*n* = 431) vs. gemcitabine (*n* = 430)	mOS 8.7 mo vs. 6.6 mo (*p <* 0.001); 1-year OS 35% vs. 22% (*p <* 0.001); 2-year OS 10% vs. 5%; 3-year OS 4% vs. 0%; mPFS 5.5 mo vs. 3.7 mo (*p <* 0.001); 1-year PFS 16% vs. 9%; response 23% vs. 7% (*p <* 0.001); mTTF 5.1 mo vs. 3.6 mo (*p <* 0.001)
Ueno and Okusaka (GEST) [69,70]	2013 and 2017	Japan and Taiwan	834	Gemcitabine plus S-1 (*n* = 275) vs. S-1 (*n* = 280) vs. gemcitabine (*n* = 277)	mOS 9.9 mo vs. 9.7 mo vs. 8.8 mo; 1-year OS 40.7% vs. 38.7% vs. 35.4%; 2-year OS 14.5% vs. 12.7% vs. 9.2%; mPFS 5.7 mo vs. 3.8 mo vs. 4.1 mo; 1-year PFS 20.3% vs. 7.2% vs. 9.1%; response 29.3% vs. 21.0% vs. 13.3%
Sudo [71]	2014	Japan	101	Gemcitabine plus S-1 (*n* = 51) vs. gemcitabine (*n* = 50)	mOS 8.6 mo vs. 8.6 mo (*p* = 0.714); mPFS 5.3 mo vs. 3.8 mo (*p* = 0.039); response 21.6% vs. 6% (*p* = 0.048)
Petrioli [72]	2015	Italy	67	Gemcitabine + capecitabine + oxaliplatin (*n* = 34) vs. gemcitabine (*n* = 33)	mOS 11.9 mo vs. 7.1 mo (*p <* 0.001); mPFS 6.8 mo vs. 3.7 mo (*p <* 0.001); 4-month control 79.4% vs. 45.4% (*p* = 0.08)
Wang [73]	2015	Taiwan	88	Gemcitabine plus erlotinib (*n* = 44) vs. gemcitabine (*n* = 44)	mOS 7.2 mo vs. 4.4 mo (*p <* 0.001); mPFS 3.8 mo vs. 2.4 mo (*p <* 0.001); control 64% vs. 25% (*p <* 0.001)
Lee [74]	2017	Korea	214	Gemcitabine + capecitabine (830 mg/m2 bid po 3/4 weeks) (*n* = 103) vs. gemcitabine (standard dose) (*n* = 101)	mOS 10.3 mo vs. 7.5 mo (*p* = 0.06); mPFS 6.2 mo vs. 5.3 mo (*p* = 0.08); response 43.7% vs. 17.6% (*p* = 0.001)

**Table 4:** Summary of randomized controlled trials concerning chemoradiation in patients with locally advanced pancreatic carcinomas.

Trial	Year	Country/Region	N	Regimens	Outcomes
Chauffert [77] (2000-01 FFCD/SFRO)	2008	France	119	CRT (60 Gy, 5-FU/cisplatin) plus maintenance gemcitabine chemotherapy (*n* = 59) vs. gemcitabine chemotherapy (*n* = 60)	mOS 8.6 mo vs. 13 mo (*p* = 0.03); 1-year OS 32% vs. 53%; 1-year PFS 14% vs. 32%
Loehrer [78]	2011	USA	74	CRT (50.4 Gy, gemcitabine) (*n* = 34) vs. gemcitabine chemotherapy (*n* = 37)	mOS 11.1 mo vs. 9.2 mo (*p* = 0.017); mPFS 6.0 mo vs. 6.7 mo; response 6% vs. 5%; stable 68% vs. 35%
Hammel (LAP-07) [79]	2016	International	449	gemcitabine (*n* = 223) vs. gemcitabine + erlotinib (*n* = 219)	From first randomization, mOS 13.6 mo vs. 11.9 mo (*p* = 0.09); mPFS 7.8 mo vs. 6.5 mo (*p* = 0.26)
			269	chemotherapy same as previously for 2 mo (*n* = 136) vs. CRT (54 Gy, capecitabine) (*n* = 133)	From first randomization, mOS 16.5 mo vs. 15.2 mo (*p* = 0.83); mPFS 8.4 mo vs. 9.9 mo (*p* = 0.06); local progression 46% vs. 32% (*p* = 0.03)
Li [80]	2003	Taiwan	34	CRT (50.4~61.2 Gy, gemcitabine) (*n* = 18) vs. CRT (50.4~61.2 Gy, 5-FU) (*n* = 16), both followed by gemcitabine chemotherapy	mOS 14.5 mo vs. 6.7 mo (*p* = 0.027); 1-y OS 56% vs. 31%; 2-y OS 15% vs. 0%; mTTP 7.1 mo vs. 2.7 mo (*p* = 0.019); response 50% vs. 12.5% (*p* = 0.005)
Wilkowski [81]	2009	Germany	95	CRT (50 Gy, 5-FU) (*n* = 30) vs. CRT (50 Gy, gemcitabine/cisplatin) (*n* = 32) vs. CRT (50 Gy, gemcitabine/cisplatin) followed by gemcitabine/cisplatin chemotherapy (*n* = 31)	mOS 9.6 mo vs. 9.3 mo vs. 7.3 mo (*p* = 0.61); 9-mo OS 58% vs. 52% vs. 45%; mPFS 4.0 mo vs. 5.6 mo vs. 6.0 mo (*p* = 0.21); response 19% vs. 22% vs. 13%
Mukherjee and Hurt [82,83]	2013 and 2017	UK	114	Induction chemotherapy with gemcitabine and capecitabine for 12 weeks, if no tumor progression, then chemotherapy with gemcitabine and capecitabine for another cycle; then CRT (50.4 Gy, capecitabine) (*n* = 36) vs. CRT (50.4 Gy, gemcitabine) (*n* = 38)	mOS 17.6 mo vs. 14.6 mo (*p* = 0.185); 1-year OS 79.2% vs. 64.2%; mPFS 12.0 mo vs. 10.4 mo (*p* = 0.120); 9-mo PFS 62.9% vs. 51.4%; response (26 weeks) 23% vs. 19%

## Abbreviations

PDAC	Pancreatic ductal adenocarcinoma
EUS-FNA	Endoscopic ultrasound-guided fine needle aspiration
CRT	Chemoradiotherapy
ECOG	Eastern Co-operative Oncology Group
NK cells	Natural killer cells
APC	Antigen presenting cells
MHC	Major histocompatibility complex
PD-1	Programed cell death-1
CTLA-4	Cytotoxic T-lymphocyte associated protein-4
FAP	Fibroblast-associated protein
CAF	Cancer-associated fibroblast
GM-CSF	Granulocyte-macrophage colony-stimulating factor
PanIN	Pancreatic intraepithelial neoplasm
CDKN2A	Cyclin dependent kinase inhibitor 2A
TGF-β	Transforming growth factor β
ADEX	Aberrantly differentiated endocrine exocrine
HMT	Histone methyltransferase
HDM	Histone demethylase
HAT	Histone acetyltransferase
HDAC	Histone deacetylase
UNR	Upstream of N-ras
PIWI	P-element-induced wimpy testis
